# Fishing With (Proto)Net—A Principled Approach to Protein Target Selection

**DOI:** 10.1002/cfg.328

**Published:** 2003-10

**Authors:** Michal Linial

**Affiliations:** Department of Biological Chemistry Institute of Life Sciences The Hebrew University Jerusalem 91904 Israel

## Abstract

Structural genomics strives to represent the entire protein space. The first step towards achieving this goal is by rationally selecting proteins whose structures have not been determined, but that represent an as yet unknown structural superfamily or fold. Once such a structure is solved, it can be used as a template for modelling homologous proteins. This will aid in unveiling the structural diversity of the protein space. Currently, no reliable method for accurate 3D structural prediction is available when a sequence or a structure homologue is not available. Here we present a systematic methodology for selecting target proteins whose structure is likely to adopt a new, as yet unknown superfamily or fold. Our method takes advantage of a global classification of the sequence space as presented by ProtoNet-3D, which is a hierarchical agglomerative clustering of the proteins of interest (the proteins in Swiss-Prot) along with all solved structures (taken from the PDB). By navigating in the scaffold of ProtoNet-3D, we yield a prioritized list of proteins that are not yet structurally solved, along with the probability of each of the proteins belonging to a new superfamily or fold. The sorted list has been self-validated against real structural data that was not available when the predictions were made. The practical application of using our computational–statistical method to determine novel superfamilies for structural genomics projects is also discussed.


**Comparative and Functional Genomics**
*Comp Funct Genom* 2003; **4**: 542–548.
Published online in Wiley InterScience (www.interscience.wiley.com). **DOI:** 10.1002/cfg.328

**Conference Review**

### Fishing with (Proto)Net — a principled approach to protein target selection

Michal Linial*
*Department of Biological Chemistry, Institute of Life Sciences, The Hebrew University, Jerusalem 91904, Israel*

**Correspondence to:*
*Michal Linial, Department of Biological Chemistry, Institute of Life Sciences, The Hebrew University, Jerusalem 91904, Israel.*
*E-mail: michall@cc.huji.ac.il*

Received: 13 July 2003
Revised: 5 August 2003
Accepted: 5 August 2003

### Abstract

**Structural genomics strives to represent the entire protein space. The first step towards achieving this goal is by rationally selecting proteins whose structures have not been determined, but that represent an as yet unknown structural superfamily or fold. Once such a structure is solved, it can be used as a template for modelling homologous proteins. This will aid in unveiling the structural diversity of the protein space. Currently, no reliable method for accurate 3D structural prediction is available when a sequence or a structure homologue is not available. Here we present a systematic methodology for selecting target proteins whose structure is likely to adopt a new, as yet unknown superfamily or fold. Our method takes advantage of a global classification of the sequence space as presented by ProtoNet-3D, which is a hierarchical agglomerative clustering of the proteins of interest (the proteins in Swiss-Prot) along with all solved structures (taken from the PDB). By navigating in the scaffold of ProtoNet-3D, we yield a prioritized list of proteins that are not yet structurally solved, along with the probability of each of the proteins belonging to a new superfamily or fold. The sorted list has been self-validated against real structural data that was not available when the predictions were made. The practical application of using our computational–statistical method to determine novel superfamilies for structural genomics projects is also discussed. Copyright © 2003 John Wiley & Sons, Ltd.**

**Keywords:** clustering; SCOP; algorithm; protein families; hierarchical classification; 3D structure

### Introduction

The goal of the structural genomics initiative is to provide a description of the structural protein space. Expanding the coverage of the structural fold space will have impact on biomedical research, including drug development [4, 8, 9, 28].

Currently, over 120 000 proteins are archived in the Swiss-Prot database and about 800 000 in the TrEMBL database (as at June, 2003). Still, the number of protein structures that are being solved to high resolution by X-ray and NMR technologies is substantially smaller. Solving a protein structure at a high resolution is a tedious multi-stepped task with some unpredicted failures along the way. Thus, choosing the correct set of proteins is critical [32]. From a sequence perspective, two proteins sharing about 35% (or more) identical amino acids will probably adopt a similar structural fold and thus, their structures can be modelled with reasonable accuracy. A good prediction for protein structure for unsolved proteins relies on the availability of a rich archive of templates for modelling [2]. Although the number of solved domains has increased significantly recently, and despite the constant effort to discover new superfamilies and folds, only a small fraction (<5%) of newly solved structures have been identified as new folds. It is clear that selecting targets with a high success rate for structural determination is a primary goal. In

Copyright © 2003 John Wiley & Sons, Ltd.

order to reach this goal, the structural community must first select target proteins that have a high probability of belonging to new superfamilies and folds [9,10,21,25,26]. Due to the enhanced pace of new structural determination, a constant update of the target list becomes essential for coping with the dynamic nature of structural genomics research. One should recall that even when a new structure is solved, the prediction of its function is still a non-trivial task [18,19,27].

### Estimation of the complexity of the protein space

Two large databases, Swiss-Prot and TrEMBL (**www.expasy.ch/sprot/**), accumulate the currently annotated proteins and potentially translated ORFs, respectively. As protein sequences accumulate, many of them already have homologues in the current database [13]. Thus, reducing the number of individual proteins to a condensed set of protein families is a step towards reconstruction of the protein space [12,22]. The number of proteins that need to be structurally solved is tightly linked to the degree of compaction of proteins into families [3,7].

As proteins not sharing any significant sequence similarity may still adopt the same structural fold, the information that is extracted from sequence homology search cannot satisfy structural inference. Several global efforts to develop methods to maximize the information from sequence towards a fold assignment have been conducted [24]. In one study, only about a quarter of the ORFs from 20 complete proteomes were assigned to known structural folds [17]. A systematic estimation of the number of structures needed to model all currently known protein families reveals that the coverage based on actual amino acids is only 10% of the entire protein mass [34]. Based on this estimation, about 16 000 coordinately selected targets will suffice to cover about 90% of all families currently known. Along this line, the remaining 10% contains most singletons or proteins that belong to small remote families. There is a more optimistic view [15], according to which the current level of protein coverage by structural models ranges between 30% and 60% for eukaryotes and prokaryotes, respectively. The conclusion from such studies is that a major portion of most proteomes still remains to be structurally explored [14]. Based on the current structural databases and the number of known homologous proteins, the number of different folds in the entire structural space (for soluble globular proteins) is estimated to range between 700 and 2000 folds [3,11]. However, without a coordinated effort for selecting targets, we expect that certain folds will become over-represented in time, while others will not be selected at all [6].

All high-resolution 3D structures are collected in the Protein Data Bank (PDB) archive that serves as a repository of all 3D structures (**www.rcsb.org/pdb**) [5]. Currently (July 2003) over 19 500 proteins are included in the main database of 3D biological macromolecular structure data (there are ~21 500 entries, including nucleic acids and carbohydrates). This collection consists of some 2000–2500 non-redundant protein structures. Several databases are available for classifying all structures to non-redundant representatives and to structurally related groups [1,30]. One such classification is provided by SCOP (**http://scop.mrc-lmb.cam.ac.uk/scop**). SCOP is a hierarchical classification of all known structural domains derived from the PDB [29] based on semi-automatic methods supported by human refinement.

The number of unique structures with novel folds added each year in the last 5 years has been rather small. According to SCOP assignment, it is only 3–5% per year for new folds and about 10% for new superfamilies. Figure 1 plots the increase in information as reported from November 1997 to March 2003. While a constant increase in the number of new folds as well as new superfamilies and families is evident, the increase is very moderate in relation to new folds.

### Scaffold of the protein sequence space — ProtoNet

In recent years, attempts to describe the protein space by clustering and other classification methods have been introduced (reviewed in [16]). Most methods are based on structural, evolutionary or sequence information. The latter can be divided into methods in which the basic element for clustering is the protein itself, or alternatively its domains.

Classification of all the proteins in the Swiss-Prot database allows the construction of a *network of relatedness* among different families. We

Copyright © 2003 John Wiley & Sons, Ltd.

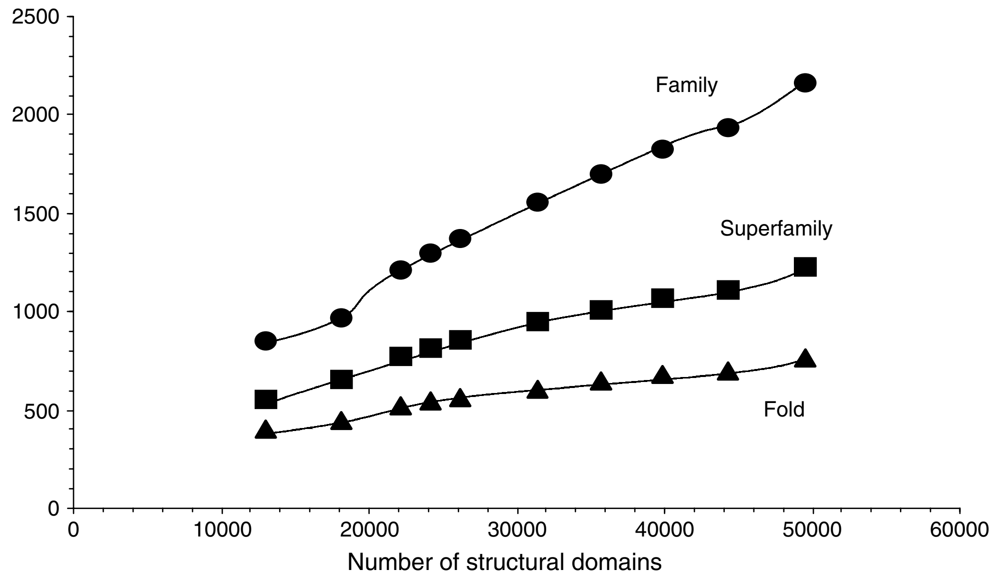

**Figure 1.** Gradual addition of solved structures to SCOP classification. SCOP major releases (from 1.37 to 1.63, total of 5.5 years) are included according to the Family, Superfamilies and Folds classification have shown that in many instances, neighbouring clusters encode biologically meaningful relations [31,35]. The notion of using a scaffold of all protein sequences to infer information on structural relatedness is the basis for our statistical–computational approach. Our approach is based on advanced hierarchical clustering algorithms using an all-against-all distances measure as the input for the algorithm. Recently, we have developed a classification method that is presented by ProtoNet (**www.protonet.cs.huji.ac.il**). In ProtoNet, all of the proteins of Swiss-Prot are considered (~120 000 proteins). The clustering process continues until most proteins are converged to only a few trees in which the leaves are the individual proteins and each node in the tree represents a cluster [33]. Altogether there are about 110 000 clusters that contain at least two proteins throughout all levels of resolution. However, toward the top of the hierarchy, clusters are merged to a few large trees and only <0.5% of the proteins remain singletons.

ProtoNet-3D is a new database that we have created that includes Swiss-Prot proteins combined with sequences archived in the PDB. This database includes over 150 000 proteins (~100 000 after reducing all expected redundancy). A clustering process was initiated on ProtoNet-3D to create hierarchical trees in which all solved structural domains are marked. Inspecting ProtoNet-3D clusters from structural perspective reveals that rich structural information is captured in the ProtoNet trees. Specifically, clusters for proteins that consist of single domains are generally pure in terms of their structural family definition, while clusters composed of multi-domain proteins often contain more than one structural entity (Shachar and Linial, in preparation). The notion of using the roadmap of all protein sequences to infer information on structural relatedness is the basis for our statistical–computational approach.

### Navigating in the roadmap of sequence and structure

One way to learn about the correspondence between structure- vs. sequence-based classifications is to discuss the coverage of ProtoNet-3D via SCOP definition. In other words, how well proteins associated with a SCOP family are included in a sequence-based cluster vs. how well a ProtoNet cluster is, structurally speaking, pure and does not contain proteins belonging to other SCOP families. The results were based on families that contain more than one protein in our database (non-trivial families). More than half of these SCOP families are fully matched with their ProtoNet clusters.

Copyright © 2003 John Wiley & Sons, Ltd. 

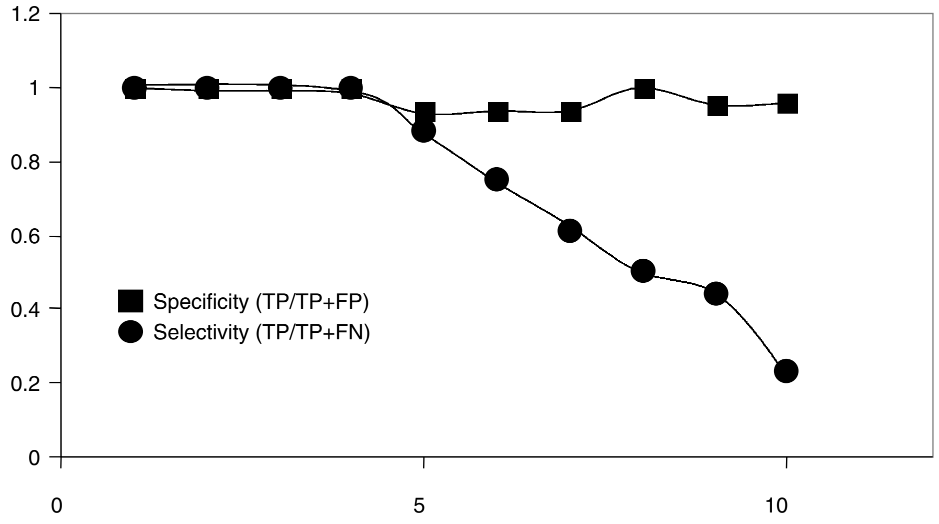

**Figure 2.** Correspondence of ProtoNet clusters with SCOP family assignment. About 750 SCOP families with at least two proteins in each are analysed. All of these families are divided into 10 bins (*x* axis) showing the level of specificity and selectivity (purity and coverage, respectively) for each of these SCOP families. About 50% of the SCOP families are in full agreement with ProtoNet clusters

For those, a sequence cluster and a structural family correspond to each other perfectly (Figure 2). Our selection for target for structural determination relies on such hidden structural information within the sequence roadmaps.

### A ranking method for target selection

Based on inspecting the correspondence between sequence and structure maps (ProtoNet-3D), we hypothesized that distances on the graph are consistent with distances between protein structures. Naively, distances can be measured by counting the number of merging steps of a particular cluster in relation to the total merging steps that are included in ProtoNet system. Our hypothesis is that the probability of a cluster without a solved protein to belong to a new, yet unknown superfamily (or fold) correlates with the distance as reflected by the ProtoNet hierarchy. Practically, if comparing a cluster that is distant in the graph from any known structure with a cluster that is near to a known structure, the former would stand a higher chance of containing a new superfamily or fold.

For a global measure of the entire protein sequence tree, we developed a scheme for prioritizing proteins according to the probability that they belong to new superfamilies. The method is based on navigation in the roadmap of protein sequences while using all structural information that is included in ProtoNet-3D. The more distant a cluster is from a previously solved structure, the more likely it is to contain a new, yet unsolved structure. We define an intrinsic measure for a protein, called 'first-solved-ancestor' (FSA). An FSA for a protein is the first ancestor containing a solved 3D structure that is encountered by climbing up the clustering tree. The higher the location in the tree the FSA of a protein, the greater is the chance of it belonging to a new superfamily.

We present a sorted list that marks each protein with a score of its probability of belonging to new superfamily. Inspection of the top list indicates that many of the top scores in our list are membranous clusters. Those clusters include many of the ion channels, receptors, transporters and pumps. The abundance of membranous clusters in the top of our list is consistent with the very limited number of membranous proteins that have been currently solved [20]. Determining the 3D structure of membranous proteins on a large scale is beyond the reach of the current technology. Thus, despite their high score as preferred targets, from a practical consideration, they should be filtered out.

Copyright © 2003 John Wiley & Sons, Ltd.

### Validating the target list

We validated our prediction method with a 'real-world' test, using major versions of SCOP (from release 1.37 to 1.61). The rationale behind the test is that once the target list is produced, we may use all structures that were not included in the prediction and were added to the SCOP database at later stages as our unbiased test. Our structure test set was the superfamilies that *did not* appear in the SCOP release that was used for creating the sorted list and *did* appear in a newer release. As the number of records grew by almost four folds in the last 5 years (Figure 1), we can expect the validation tests to be statistically sound (for more details on the validation test see [31]).

The test case includes structures that were not available when the target list was produced. Our results were all converted to indicate the success in predicting new superfamilies by $p$-value measures. The $p$-value reflects the probability of obtaining this level of success, or better, at a random setting. We determined the optimal possible threshold in the ProtoNet-3D tree that accurately separates the proteins that belong to already known superfamilies from those representing new ones. This threshold was used to test the performance of the prediction. The FSA-based method that was used on SCOP 1.55 as the base set and SCOP 1.61 as the test set resulted in a success rate of about 80% in separating new superfamilies from known ones (Kifer, Sasson and Linial, in preparation).

### Practical considerations

Even once a list of targets is presented to the experimentalist, success in obtaining high quality 3D structure depends on many practical considerations and constraints. Not all proteins are suitable for crystallization, and some of those limitations can already be suggested by inspecting their primary sequences [17]. Practical issues, such as the level of solubility and the predicted success in producing high enough amounts, must also be taken into account. These factors can dominate the success rate of structural determination in any large-scale effort. A prediction for the success rate of structural determination has been complicated by the fact that many proteins do not function alone, but rather with additional entities. These entities may be another protein, nucleic acids, co-factors or small molecule inhibitors. If we consider multi-domain protein, it may be better expressed as a whole protein to improve stability and solubility of the expressed protein. Preferably, a protein should be chosen as a target for a structural genomics project based on a combination of theoretical and practical predictions for success.

To assist the selection of appropriate targets in the scope of structural genomics projects, we designed an interactive website called ProTarget. This site is designed to present potential targets for structural determination. To this end, filtering criteria to narrow down the number of targets according to biological and other practical considerations are included. Such filters include the length of the selected proteins, the source of the proteins (organisms) within the cluster, the number of proteins within the cluster and more. In addition, experimentalists should consider their methodology of choice, either NMR or X-ray (or a combination of the two). Once decided, the target list may be restricted to proteins that fit constraints on protein length, as defined by the user. The list of target proteins is available at **http://www.protarget.cs.huji.ac.il**.

The target list that is produced using the FSA ranking method is very sensitive to the dynamic nature of new structures that are constantly being solved. A dynamic iterative query indicates the effect of solving new structures on the other clusters in the map. In the new version of ProTarget, the user will have the option of marking any cluster as 'solved' and thus monitoring the effect of solving proteins on the other clusters. Following such user-dependent iterations, new lists are calculated based on a revised set of clusters. The ability to navigate iteratively in the roadmap of protein clusters may be used to scan those proteins that, once solved, will have maximal impact on the rest of the map.

### Perspective and future directions

In discussing target selection, it is extremely valuable to define which of the proteins with currently unsolved structure can be modelled to a high level by other solved structures. The challenge of structural prediction methods is to determine whether a protein may have a new superfamily or fold. Proteins sharing more than 30–35% identity in their

Copyright © 2003 John Wiley & Sons, Ltd. 

sequence are candidates for accurate modelling. We assume that for most of them, ProtoNet supports high quality predictions and robust protein family definition.

Due to a worldwide effort in structural genomics, protein structures are being deposited in the PDB archive at an accelerated pace [5]. Consequently, an essential component of any proposed target list is a method that copes with these advances. Setting a framework that allows measuring the success in discovering new structural superfamilies and folds is an intriguing one. With such a setting, the structural community may test the relevance of a systematic approach for target selection. A competing brute-force approach calls for experimentally attempting all possible proteins that are yet unsolved. At the moment, it is too early to estimate the contribution of each of these two extreme approaches that aim to discover the entire structural space.

### Acknowledgements

This review summarizes the contributions of Ilona Kifer, Ori Shachar, Ori Sasson and Noam Kaplan. We would like to thank Elon Portugaly and Nati Linial for valuable discussions and suggestions throughout this study. We thank Sharone Tayar for critical reading and suggestions. This study is supported by an NIH grant (for the Center for Eukaryotic Structural Genomics, CESG, Madison, USA). The Sudarsky Center for Computational Biology in the Hebrew University supports I. Kifer and O. Shachar.

### References

1. Al-Hashimi HM, Gorin A, Majumdar A, Gosser Y, Patel DJ. 2002. Towards structural genomics of RNA: rapid NMR resonance assignment and simultaneous RNA tertiary structure determination using residual dipolar couplings. *J Mol Biol* **318**: 637–649.
2. Baker D, Sali A. 2001. Protein structure prediction and structural genomics. *Science* **294**: 93–96.
3. Balasubramanian S, Schneider T, Gerstein M, Regan L. 2000. Proteomics of *Mycoplasma genitalium*: identification and characterization of unannotated and atypical proteins in a small model genome. *Nucleic Acids Res* **28**: 3075–3082.
4. Baumeister W, Steven AC. 2000. Macromolecular electron microscopy in the era of structural genomics. *Trends Biochem Sci* **25**: 624–631.
5. Berman HM, Bhat TN, Bourne PE, *et al*. 2000. The Protein Data Bank and the challenge of structural genomics. *Nature Struct Biol* **7**(suppl): 957–959.
6. Brenner SE, Barken D, Levitt M. 1999. The PRESAGE database for structural genomics. *Nucleic Acids Res* **27**: 251–253.
7. Brenner SE. 2000. Target selection for structural genomics. *Nature Struct Biol* **7**(suppl): 967–969.
8. Buchanan SG. 2002. Structural genomics: bridging functional genomics and structure-based drug design. *Curr Opin Drug Discov Devel* **5**: 367–381.
9. Burley SK, Almo SC, Bonanno JB, *et al*. 1999. Structural genomics: beyond the human genome project. *Nature Genet* **23**: 151–157.
10. Burley SK. 2000. An overview of structural genomics. *Nature Struct Biol* **7**(suppl): 932–934.
11. Chothia C. 1992. Proteins. One thousand families for the molecular biologist. *Nature* **357**: 543–544.
12. Eisenstein E, Gilliland GL, Herzberg O, *et al*. 2000. Biological function made crystal clear — annotation of hypothetical proteins via structural genomics. *Curr Opin Biotechnol* **11**: 25–30.
13. Frishman D. 2002. Knowledge-based selection of targets for structural genomics. *Protein Eng* **15**: 169–183.
14. Gerstein M, Hegyi H. 1998. Comparing genomes in terms of protein structure: surveys of a finite parts list. *FEMS Microbiol Rev* **22**: 277–304.
15. Gough J, Chothia C. 2002. SUPERFAMILY: HMMs representing all proteins of known structure. SCOP sequence searches, alignments and genome assignments. *Nucleic Acids Res* **30**: 268–272.
16. Heger A, Holm L. 2000. Towards a covering set of protein family profiles. *Prog Biophys Mol Biol* **73**: 321–337.
17. Hegyi H, Lin J, Greenbaum D, Gerstein M. 2002. Structural genomics analysis: characteristics of atypical, common, and horizontally transferred folds. *Proteins* **47**: 126–141.
18. Irving JA, Whisstock JC, Lesk AM. 2001. Protein structural alignments and functional genomics. *Proteins* **42**: 378–382.
19. Jackson RM, Russell RB. 2001. Predicting function from structure: examples of the serine protease inhibitor canonical loop conformation found in extracellular proteins. *Comput Chem* **26**: 31–39.
20. Jones DT, Taylor WR. 1998. Towards structural genomics for transmembrane proteins. *Biochem Soc Trans* **26**: 429–438.
21. Kim SH. 1998. Shining a light on structural genomics. *Nature Struct Biol* **5**(suppl): 643–645.
22. Kim SH. 2000. Structural genomics of microbes: an objective. *Curr Opin Struct Biol* **10**: 380–383.
23. Koonin EV, Wolf YI, Aravind L. 2000. Protein fold recognition using sequence profiles and its application in structural genomics. *Adv Protein Chem* **54**: 245–275.
24. Lindahl E, Elofsson A. 2000. Identification of related proteins on family, superfamily and fold level. *J Mol Biol* **295**: 613–625.
25. Linial M, Yona G. 2000. Methodologies for target selection in structural genomics. *Prog Biophys Mol Biol* **73**: 297–320.
26. Mallick P, Goodwill KE, Fitz-Gibbon S, Miller JH, Eisenberg D. 2000. Selecting protein targets for structural genomics of *Pyrobaculum aerophilum*: validating automated fold assignment methods by using binary hypothesis testing. *Proc Natl Acad Sci USA* **97**: 2450–2455.
27. Marti-Renom MA, Stuart AC, Fiser A, *et al*. 2000. Comparative protein structure modeling of genes and genomes. *Ann Rev Biophys Biomol Struct* **29**: 291–325.
28. Mittl PR, Grutter MG. 2001. Structural genomics: opportunities and challenges. *Curr Opin Chem Biol* **5**: 402–408.

Copyright © 2003 John Wiley & Sons, Ltd. 

29. Murzin AG, Bateman A. 2001. CASP2 knowledge-based approach to distant homology recognition and fold prediction in CASP4. *Proteins* **5**(suppl): 76–85.
30. Pearl FM, Martin N, Bray JE, *et al*. 2001. A rapid classification protocol for the CATH domain database to support structural genomics. *Nucleic Acids Res* **29**: 223–227.
31. Portugaly E, Kifer I, Linial M. 2002. Selecting targets for structural determination by navigating in a graph of protein families. *Bioinformatics* **18**: 899–907.
32. Sali A. 1998. 100 000 protein structures for the biologist. *Nature Struct Biol* **5**: 1029–1032.
33. Sasson O, Linial N, Linial M. 2002. The metric space of proteins-comparative study of clustering algorithms. *Bioinformatics* **18**(suppl 1): 14–21.
34. Vitkup D, Melamud E, Moult J, Sander C. 2001. Completeness in structural genomics. *Nature Struct Biol* **8**: 559–566.
35. Yona G, Linial N, Linial M. 1999. ProtoMap: automatic classification of protein sequences, a hierarchy of protein families, and local maps of the protein space. *Proteins* **37**: 360–378.

Copyright © 2003 John Wiley & Sons, Ltd. 

## References

[PDF_00438] Al-Hashimi Hashim M., Gorin Andrey, Majumdar Ananya, Gosser Yuying, Patel Dinshaw J. (2002). Towards structural genomics of RNA: rapid NMR resonance assignment and simultaneous RNA tertiary structure determination using residual dipolar couplings.. J Mol Biol.

[PDF_00443] Baker D., Sali A. (2001). Protein structure prediction and structural genomics.. Science.

[PDF_00445] Balasubramanian S., Schneider T., Gerstein M., Regan L. (2000). Proteomics of Mycoplasma genitalium: identification and characterization of unannotated and atypical proteins in a small model genome.. Nucleic Acids Res.

[PDF_00449] Baumeister W., Steven A. C. (2000). Macromolecular electron microscopy in the era of structural genomics.. Trends Biochem Sci.

[PDF_00452] Berman H. M., Bhat T. N., Bourne P. E., Feng Z., Gilliland G., Weissig H., Westbrook J. (2000). The Protein Data Bank and the challenge of structural genomics.. Nat Struct Biol.

[PDF_00455] Brenner S. E., Barken D., Levitt M. (1999). The PRESAGE database for structural genomics.. Nucleic Acids Res.

[PDF_00458] Brenner S. E. (2000). Target selection for structural genomics.. Nat Struct Biol.

[PDF_00460] Buchanan Sean G. (2002). Structural genomics: bridging functional genomics and structure-based drug design.. Curr Opin Drug Discov Devel.

[PDF_00463] Burley S. K., Almo S. C., Bonanno J. B., Capel M., Chance M. R., Gaasterland T., Lin D., Sali A., Studier F. W., Swaminathan S. (1999). Structural genomics: beyond the human genome project.. Nat Genet.

[PDF_00466] Burley S. K. (2000). An overview of structural genomics.. Nat Struct Biol.

[PDF_00468] Chothia C. (1992). Proteins. One thousand families for the molecular biologist.. Nature.

[PDF_00470] Eisenstein E., Gilliland G. L., Herzberg O., Moult J., Orban J., Poljak R. J., Banerjei L., Richardson D., Howard A. J. (2000). Biological function made crystal clear - annotation of hypothetical proteins via structural genomics.. Curr Opin Biotechnol.

[PDF_00474] Frishman Dmitrij (2002). Knowledge-based selection of targets for structural genomics.. Protein Eng.

[PDF_00476] Gerstein M., Hegyi H. (1998). Comparing genomes in terms of protein structure: surveys of a finite parts list.. FEMS Microbiol Rev.

[PDF_00479] Gough Julian, Chothia Cyrus (2002). SUPERFAMILY: HMMs representing all proteins of known structure. SCOP sequence searches, alignments and genome assignments.. Nucleic Acids Res.

[PDF_00483] Heger A., Holm L. (2000). Towards a covering set of protein family profiles.. Prog Biophys Mol Biol.

[PDF_00485] Hegyi Hedi, Lin Jimmy, Greenbaum Dov, Gerstein Mark (2002). Structural genomics analysis: characteristics of atypical, common, and horizontally transferred folds.. Proteins.

[PDF_00488] Irving J. A., Whisstock J. C., Lesk A. M. (2001). Protein structural alignments and functional genomics.. Proteins.

[PDF_00490] Jackson R. M., Russell R. B. (2001). Predicting function from structure: examples of the serine protease inhibitor canonical loop conformation found in extracellular proteins.. Comput Chem.

[PDF_00494] Jones D. T., Taylor W. R. (1998). Towards structural genomics for transmembrane proteins.. Biochem Soc Trans.

[PDF_00500] Koonin E. V., Wolf Y. I., Aravind L. (2000). Protein fold recognition using sequence profiles and its application in structural genomics.. Adv Protein Chem.

[PDF_00503] Lindahl E., Elofsson A. (2000). Identification of related proteins on family, superfamily and fold level.. J Mol Biol.

[PDF_00506] Linial M., Yona G. (2000). Methodologies for target selection in structural genomics.. Prog Biophys Mol Biol.

[PDF_00508] Mallick P., Goodwill K. E., Fitz-Gibbon S., Miller J. H., Eisenberg D. (2000). Selecting protein targets for structural genomics of Pyrobaculum aerophilum: validating automated fold assignment methods by using binary hypothesis testing.. Proc Natl Acad Sci U S A.

[PDF_00513] Martí-Renom M. A., Stuart A. C., Fiser A., Sánchez R., Melo F., Sali A. (2000). Comparative protein structure modeling of genes and genomes.. Annu Rev Biophys Biomol Struct.

[PDF_00516] Mittl P. R., Grütter M. G. (2001). Structural genomics: opportunities and challenges.. Curr Opin Chem Biol.

[PDF_00520] Murzin A. G., Bateman A. (2001). CASP2 knowledge-based approach to distant homology recognition and fold prediction in CASP4.. Proteins.

[PDF_00523] Pearl F. M., Martin N., Bray J. E., Buchan D. W., Harrison A. P., Lee D., Reeves G. A., Shepherd A. J., Sillitoe I., Todd A. E. (2001). A rapid classification protocol for the CATH Domain Database to support structural genomics.. Nucleic Acids Res.

[PDF_00526] Portugaly Elon, Kifer Ilona, Linial Michal (2002). Selecting targets for structural determination by navigating in a graph of protein families.. Bioinformatics.

[PDF_00529] Sali A. (1998). 100,000 protein structures for the biologist.. Nat Struct Biol.

[PDF_00534] Vitkup D., Melamud E., Moult J., Sander C. (2001). Completeness in structural genomics.. Nat Struct Biol.

[PDF_00536] Yona G., Linial N., Linial M. (1999). ProtoMap: automatic classification of protein sequences, a hierarchy of protein families, and local maps of the protein space.. Proteins.

